# Continuous Production of Biogenic Magnetite Nanoparticles by the Marine Bacterium *Magnetovibrio blakemorei* Strain MV-1^T^ with a Nitrous Oxide Injection Strategy

**DOI:** 10.3390/md20110724

**Published:** 2022-11-18

**Authors:** Tarcisio Correa, Mateus G. Godoy, Dennis A. Bazylinski, Fernanda Abreu

**Affiliations:** 1 Instituto de Microbiologia Paulo de Góes, Universidade Federal do Rio de Janeiro, Rio de Janeiro 21941-902, Brazil; 2 School of Life Sciences, University of Nevada, Las Vegas, NV 89154-4004, USA

**Keywords:** magnetotactic bacteria, magnetosomes, chemostat, fed-batch

## Abstract

Magnetotactic bacteria (MTB) produce magnetosomes, which are membrane-embedded magnetic nanoparticles. Despite their technological applicability, the production of magnetite magnetosomes depends on the cultivation of MTB, which results in low yields. Thus, strategies for the large-scale cultivation of MTB need to be improved. Here, we describe a new approach for bioreactor cultivation of *Magnetovibrio blakemorei* strain MV-1^T^. Firstly, a fed-batch with a supplementation of iron source and N_2_O injection in 24-h pulses was established. After 120 h of cultivation, the production of magnetite reached 24.5 mg∙L^−1^. The maximum productivity (16.8 mg∙L^−1^∙day^−1^) was reached between 48 and 72 h. However, the productivity and mean number of magnetosomes per cell decreased after 72 h. Therefore, continuous culture in the chemostat was established. In the continuous process, magnetite production and productivity were 27.1 mg∙L^−1^ and 22.7 mg∙L^−1^∙day^−1^, respectively, at 120 h. This new approach prevented a decrease in magnetite production in comparison to the fed-batch strategy.

## 1. Introduction

Magnetic nanomaterials are among the most versatile tools available for manufacturing, medicine, and the environmental sciences. Magnetic nanoparticles are continuously making their way into the market in the form of recyclable nanocatalysts, controllable drug nanocarriers, and ultrasensitive nanosensors [1,2]. The nanomagnets used in these refined approaches must be produced with suitable quality and adequate quantity to assure effectiveness and safety. For this reason, the fabrication process of nanomagnets needs to be developed [3].

Several wet and dry chemical methods are characterized in existing literature for the synthesis of magnetic nanoparticles [4]. However, only a few have been adapted to a large scale [3]. Thermal decomposition and co-precipitation are the most explored chemical processes and often result in productions larger than the gram scale [3]. Simeonidis and colleagues [5] designed a continuous flow stirred tank process (5 L working volume) to synthesize iron oxide and sulfide nanoparticles by alkaline co-precipitation of Fe^2+^ and Fe^3+^. This method resulted in productions of 0.33 kg/h for iron oxide and 1 kg/h for iron sulfide nanoparticles, with mean diameters of 18 and 20 nm, respectively [5]. However, the nanoparticles presented some aggregation and relatively low shape uniformity. Additionally, such processes generate a considerable volume of highly alkaline aqueous waste, which demands treatment steps before discharge. Park and colleagues designed the production of magnetite nanoparticles with very narrow (<5%) size dispersion, with a yield of 40 g per reaction [6]. The process was based on the thermal decomposition of iron oleate in boiling solvents. A similar process has been associated with chemical emissions, impacting air and water [7].

While the end-use toxicity of nanoparticles is already subject to international regulations [1], environmental impacts related to their manufacture are still not well examined [7]. In the scenario of a growing demand for magnetic nanoparticles, cleaner processes are necessary to avoid ecotoxic emissions and high energy consumption. In this sense, biotechnological syntheses of nanoparticles are considered greener alternatives to processes based only on chemicals [8]. As an example of this alternative, plant extracts have been used as precipitating and stabilizing agents to synthesize the iron oxide nanocatalysts used in the degradation of dyes [9]. Microbial syntheses of various metal oxides and sulfide nanoparticles are described in the literature [8].

Perhaps the most explored sustainable biogenic magnetic nanoparticles are produced by magnetotactic bacteria (MTB) [10]. These microorganisms are prokaryotes from different taxa whose ability to orient along magnetic field lines is conferred by the magnetosomes’ intracellular chains [11]. A set of genes organized in clusters (the so-called magnetosome gene cluster) are responsible for the finely controlled biomineralization process [12]. As a result, magnetosome nanocrystals present well-defined shapes and narrow-size distribution within a single species [13]. The single magnetic domain crystal may be either magnetite or greigite, depending upon the magnetotactic species [10]. Because magnetosomes synthesis occurs inside vesicles formed by invaginations of the cytoplasmic membrane, each magnetosome remains enveloped by the lipid bilayer [10]. This natural coating is responsible for some advantages over synthetic nanoparticles: i. it contains amino groups from embedded proteins, which are available for covalent cross-linking with functional molecules (e.g., drugs, antibodies, etc.); ii. it possesses a net negative charge, which allows electrostatic interaction with functional positively charged polymers; iii. it enables magnetosomes with colloidal stability in aqueous media; iv. it dispenses additional coating steps, which are usually required for synthetic nanoparticles and, thus, significantly reduces final material costs [14,15].

These unique characteristics make magnetosomes innovative tools in health and technology [15]. Due to their responsiveness to permanent and alternating magnetic fields, magnetosomes have been used in vitro to deliver anti proliferative drugs and the destruction of tumors by hyperthermia [15]. Other applications include enzyme and antibody immobilization, contrasting in magnetic resonance imaging, and cell sorting [15]. However, due to the fastidious growth of MTB and low yield of magnetosomes during cultivation, obtaining these biologically friendly nanoparticles is still challenging, and their application is hindered [15].

Similar to synthetic nanoparticles, the production of magnetosomes in large quantities are necessary to meet industrial demands. Since the publication of the work by Heyen and Schuler [16], in which *Magnetospirillum* growth media has been optimized for bioreactor cultivation under oxygen limitation, numerous strategies to improve the production and productivity of magnetosomes have been designed [16,17,18,19]. Those strategies included modifications in media composition and culture as well as physical and chemical incubation conditions. To our knowledge, the cultivation of MTB in bioreactors has been conducted in batch [16], fed-batch [18,19], and semicontinuous modes [20]. The majority of these bioprocesses are designed for the growth of *Magnetospirillum* genus, whose cells produce cuboctahedral magnetosomes. To date, only one report of bioreactor cultivation of *Magnetovibrio blakemorei* strain MV-1^T^ has been made [21]. Magnetosomes from *Mv. blakemorei* strain MV-1^T^ display a prismatic shape and are larger (60 nm, average length) than those of *Magnetospirillum magneticum* strain AMB-1 (45 nm, average length) and *Magnetospirillum gryphiswaldense* strain MSR-1 (33 nm, average length). Their larger volume and surface area [21,22] presumably leads to a greater molecule loading of functional molecules during surface modification [21].

As a hurdle in cultivation of MTB, spontaneous nonmagnetic mutants are often found in the stationary growth of *Ms. gryphiswaldense* strain MSR-1 [23,24]. The inability to produce magnetosomes in those cells is due to deletions in different sites of the magnetosome gene cluster [24]. In *Mv. blakemorei* strain MV-1^T^, spontaneous non-magnetic mutant cells were also found in the culture [25]. These mutants did not produce several proteins present in the wild-type strain, and they lack the iron uptake system necessary for biomineralization [25]. When growing in a bioreactor, it may be a good strategy to avoid the stationary phase in order to prevent the loss of magnetosome synthesis ability by cells. To keep cells growing in a steady-state corresponding to exponential growth, it is necessary to have continuous growth, maintaining growth at a given rate indefinitely (e.g., maximum growth rate). For this, a fresh, sterile medium is inserted in the reactor vessel while the spent medium with metabolites and cell debris is being removed [26].

In this work, we execute the first reported, to date, cultivation of the marine magnetotactic vibrio *Mv. blakemorei* strain MV-1^T^ in continuous mode, using a chemostat strategy, where log phase conditions were maintained constant, including high-nutrient concentration and high magnetosome-producing cell density. To achieve this, we first carried out a fed-batch fermentation with supplementation of an iron source and N_2_O–the main final electron acceptor in microaerobic/anaerobic growth. Afterwards, we set up a gas injection regime to provide a sufficient input of N_2_O during cell growth. Lastly, we developed a chemostat continuous culture in which the productivity of magnetosomes was kept high and constant, diminishing the occurrence of late-growth-phase non-magnetic mutants. During all cultivation experiments, we performed measurements of succinate, nitrogen, and iron (II) concentrations in medium to monitor nutrient consumption and prevent depletion of carbon, nitrogen, and iron. The present paper illustrates the potential of chemostat strategy for the mass cultivation of MTB. This is especially important given the practical difficulties imposed by MTB cultivation (e.g., complex media, low productivity, etc.), as processes with longer high-productivity periods might be obtained from a single inoculum.

## 2. Results

### 2.1. Fed-Batch

**Figure 1:**
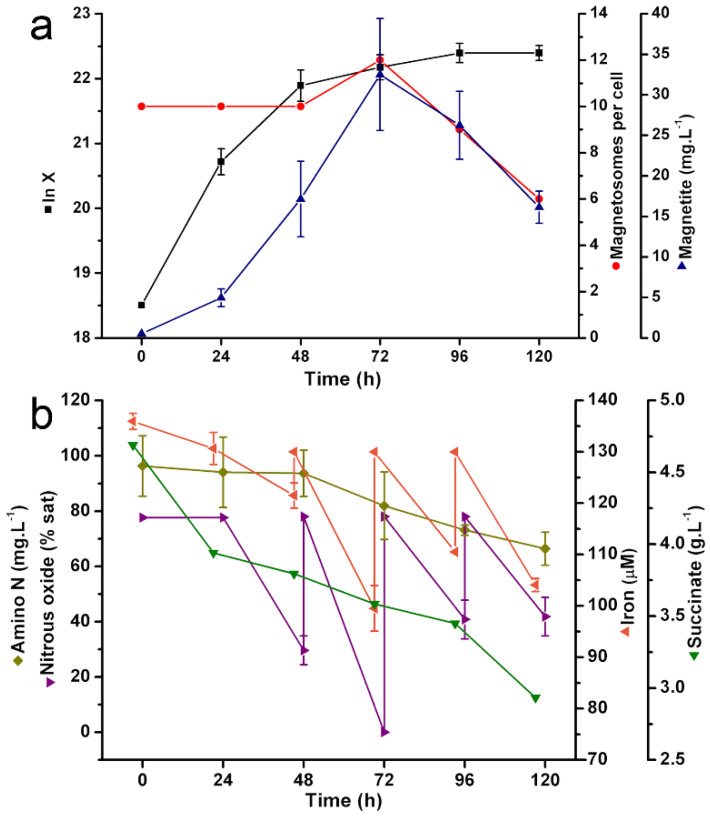
Growth kinetics of *Mv. blakemorei* strain MV-1^T^ and magnetosome formation in fed batch in a 5-L bioreactor. (**a**) Cell growth expressed in natural logarithm of cell density (ln X), number of magnetosomes per cell and global magnetite concentration. (**b**) Concentration (percent) of available iron (Fe^2+^), amino nitrogen, succinate and nitrous oxide (N_2_O) in the growth medium. The concentrations of Fe^2+^ and N_2_O were adjusted to their initial levels in the medium by injections of the respective component at 24-h intervals.

**Figure A1:**
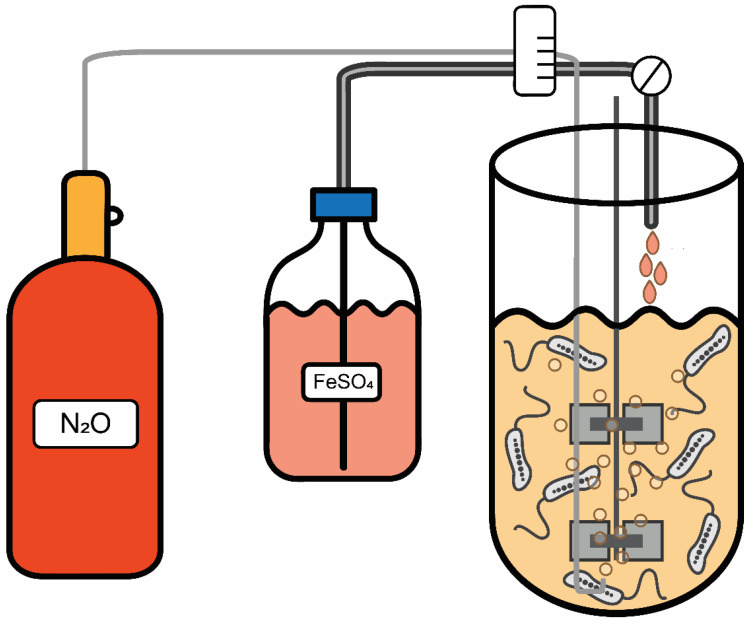
Experimental design of fed-batch cultivation used in this study. A sterile 10 mM FeSO4 solution is constantly added to the growth medium each 24 h in a volume calculated to reestablish original Fe^2+^ concentration (100 µM). N_2_O, the final electron acceptor for the cultivated strain MV-1^T^, is bubbled into liquid medium until saturation. mechanical stirring. For convenience, pH corrective solutions, and temperature and pH probes are omitted.

In our fed-batch experiments (Figure A1), *Mv. blakemorei* strain MV-1^T^ growth was observed up to 120 h, with the highest specific growth rate observed between 24 h and 48 h (μ_max_ = 0.05 h^−1^, Figure 1a). However, the mean number of magnetosomes per cell showed its maximum value at 72 h, decreasing from this time on (Figure 1a and Table 1). Magnetite production, whose maximum value reached 72 h (*p* = 32.5 mg∙L^−1^, Table 1), decreased in subsequent intervals due to the decrease in the production of magnetosomes per cell (Figure 1a). Maximum magnetite productivity was reached at 72 h (*p* = 1.65 mg∙L^−1^∙h^−1^ or 39.7 mg∙L^−1^∙day^−1^, Table 1).

Consumption of N_2_O and Fe^2+^ was more intense between 48 and 72 h. In the latter time, the average magnetosome number per cell was the highest (11 per cell). Three pulses of both N_2_O and Fe^2+^ were needed to reach their initial concentration (Figure 1b). N_2_O and Fe^2+^ supplementation increased both the production and productivity of magnetite at 72 h (32.5 mg∙L^−1^∙day^−1^ and 39.7 mg∙L^−1^, respectively); production, productivity and average number of magnetosomes per cell decreased from this time point (reaching 16.1 mg∙L^−1^, 12.6 mg∙L^−1^∙day^−1^ and 6 magnetosomes per cell, respectively; Figure 1a). Succinate was consumed throughout growth, and its concentration at 120 h was 62% of that at the time of inoculation (Figure 1b).

Carbon and nitrogen were consumed in more even rates throughout the process (Figure 1b). Consumption of nitrogen seems to be less correlated to magnetite synthesis than those of N_2_O, Fe^2+^ and carbon. Hence, there is an indication that nitrogen demand was larger for cell growth and division than for magnetosomes synthesis. Furthermore, both carbon and nitrogen were present in relative abundance in medium throughout the batch, with 40 and 70% of their initial concentration remaining in medium at 96 h of cultivation (Figure 1b). Thus, these essential nutrients were not limiting for cell growth and magnetosome production during early cultivation times.

Considering that pH substantially impacts both culture growth and magnetosome formation, our experimental set up (Figure A1) relied on a strict pH control provided by an automated built-in system that injects either sterile NaOH or HCl in response to pH variations. During fed-batch, pH was kept within the range 7.0 ± 0.2, even after the supplementation of acidic FeSO_4_ solution and N_2_O.

### 2.2. Nitrous Oxide Mass Transfer

**Figure 2:**
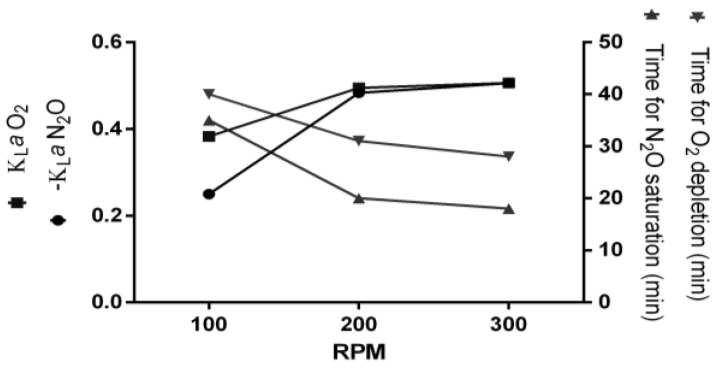
Mass transfer coefficients (kLa) of N_2_O (solubilization) and O_2_ (dislocation) and time elapsed for N_2_O saturation and O_2_ depletion.

According to our results, under the conditions of agitation rate in 100 RPM with a N_2_O flow of 0.5 L∙min^−1^, it takes 35 min to complete N_2_O saturation starting from zero N_2_O and ~20% oxygen (Figure 2). However, at 200 RPM, it takes 20 min. Using 300 RPM slightly decreases the time for N_2_O saturation to 18 min (Figure 2). From consumption analysis during growth, we can see that almost 80% of N_2_O was consumed in the exponential growth phase. As continuous culture aims at extending the exponential phase conditions, we calculated a N_2_O injection regime that kept N_2_O at a level of at least 75% of saturation, considering that the consumption of 80% between 48–72 h, 25% of N_2_O would be consumed within 8 h. Thus, a regime of N_2_O injection to saturation level every 8 h would keep the concentration at least 75% of saturation during continuous cultivation. Considering the N_2_O concentration before sparging and a kLa value of 0.48 at 200 RPM, N_2_O can be replenished within 1.9 min. Thus, agitation was set to rise from 100 to 200 RPM during 5 min while N_2_O was sparged in medium to ensure total replenishment. This helped N_2_O transfer from the gas to liquid phase while agitation’s negative effect on growth was minimized.

Oxygen dislocation was also studied in mass transfer experiments. This was done to ensure anaerobic conditions, as we cannot guarantee the feed medium was completely free of oxygen. The time and agitation chosen for N_2_O injection were enough to remove all detectable oxygen from medium (Figure 2).

### 2.3. Continuous Growth

**Figure A2:**
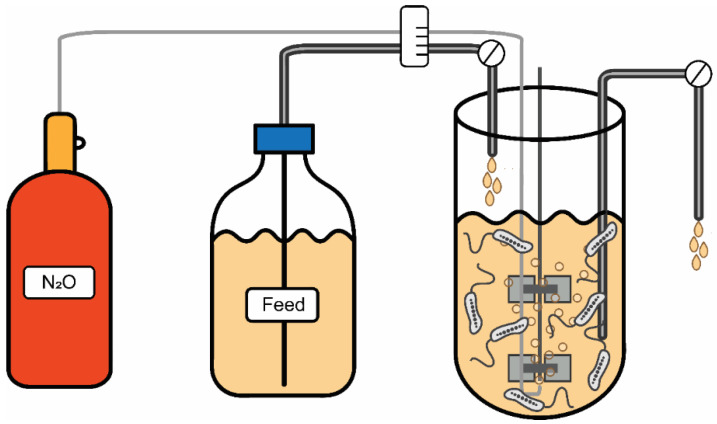
Experimental design of chemostat cultivation. Fresh growth medium is constantly added to the bioreactor vessel where bacterial growth takes place. Simultaneously, spent medium with grown cells and secondary metabolites is removed at the same pace of fresh medium inflow. N_2_O, is intermittently gassed through liquid medium under mechanical stirring.

According to the fed-batch experiments data, medium components were consumed in different rates and only the essential nutrients were examined. These limitations are hard to overcome because of the lack of deeper knowledge on bacterial nutritional demands and physiology. Thus, one strategy to support further growth of strain MV-1^T^ in bioreactors would be the addition of whole fresh culture medium (Figure A2). In fed-batch cultivation, the average number of magnetosomes per cell decreases from 72 h (Figure 1a and Table 1) while the proportion of non-magnetic cells increases (Table 1). Here, the loss of the ability to synthesize magnetosomes occurs in later times of growth, although iron continues to be replenished. This supports the idea that the decline in overall magnetite production was due to deletions in the genome region known as MGC.

In this sense, the implementation of continuous culture would help to maintain high-magnetosome producing cells, and thus directly influence the production of magnetite. Few to no non-magnetic mutants were observed in a culture of *Ms. gryphiswaldense* strain MSR-1 with inoculation before medium saturation, even after multiple uninterrupted passages [24]. After medium saturation, non-magnetotactic mutants reached 0.5% of the total population [24].

Iron, amino nitrogen, and succinate are taken up from media by growing cells at different rates, as observed for batch experiments (Figure 1). Measurements during continuous culture are intended for monitoring if substrates are being kept at sufficient levels to support constant cell density, or if any of the nutrients are being depleted in a rate greater than replenishment through fresh media inlet. The latter scenario would require us to make further adjustments in our media and operational conditions (i.e., dilution rate, separate nutrient injection, etc.).

From the results of fed batch growth, the point of 72 h was chosen to initiate continuous culture and intermittent N_2_O injection based on the maximum production of magnetite. The largest Fe^2+^ and N_2_O consumption and highest magnetite concentration took place between 48 h and 72 h (Figure 1a,b). The dilution rate employed was equal to 70% of maximum growth rate (µ_max_) during the exponential phase.

**Figure 3:**
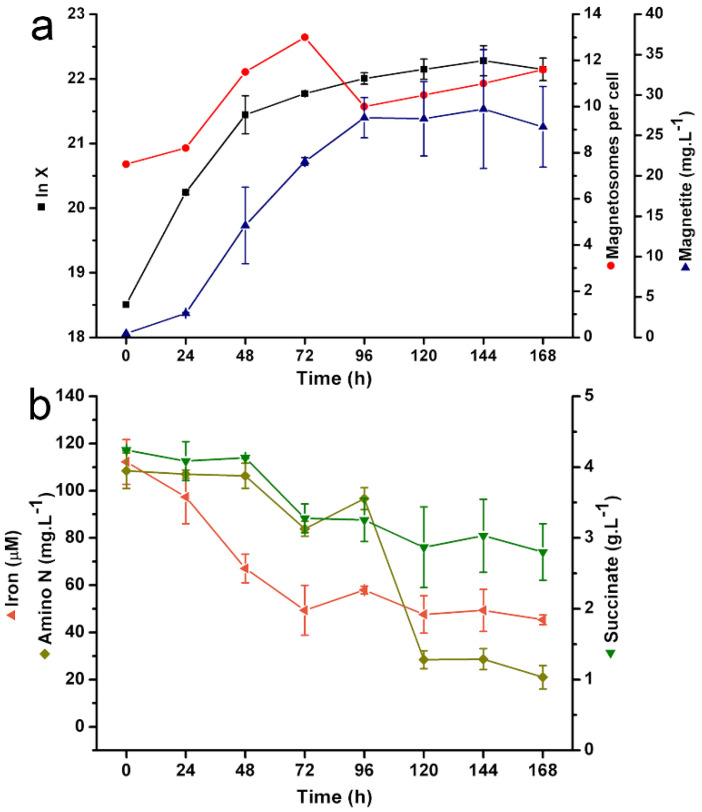
Growth kinetics of *Mv. blakemorei* strain MV-1^T^ and magnetosome formation in continuous culture (chemostat) in a 5-L bioreactor. (**a**) Cell growth expressed in natural logarithm of cell density (ln X), number of magnetosomes per cell and global magnetite concentration. (**b**) Concentration (percent) of available iron (Fe^2+^), amino nitrogen and succinate.

After the implementation of continuous culture, there was a slight raise in cell density (72–96 h–Figure 3a). Then, a slight decrease (96–120 h) in this parameter followed by stabilization (120–168 h) with small fluctuations was observed (Figure 3a). Nutrient concentrations also stabilized between 72–120 h, remaining approximately constant until the end of cultivation (Figure 3b). These phenomena characterize the onset of the steady state.

**Figure 4:**
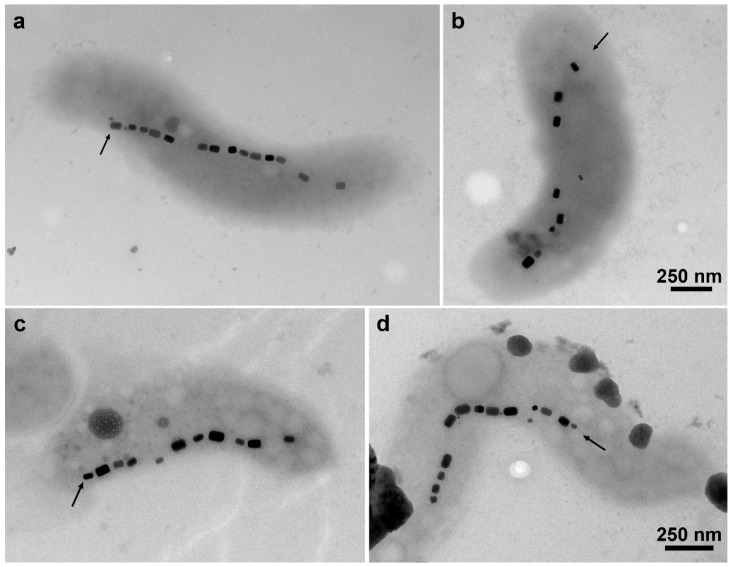
(**a**,**b**) Transmission electron microscopy of *Mv. blakemorei* strain MV-1^T^ grown in fed batch with supplementation of Fe^2+^ and N_2_O at 48 h (**a**) and 120 h (**b**). A decrease in the number of magnetosomes per cell is evident at the end of cultivation (arrows indicate magnetosomes chains). (**c**,**d**) Transmission electron microscopy of *Mv. blakemorei* strain MV-1^T^ grown in chemostat for 48 h (**c**) and 120 h (**d**). Average number of magnetosomes per cell were roughly constant throughout the cultivation (arrows indicate magnetosomes chains). Scale bar in (**b**,**d**) applies to panel (**a**,**c**), respectively.

A substantial increase in the average number of magnetosomes per cell and a sharp decrease in the proportion of non-magnetic cells (Figure 3a and Figure 4c,d, Table 1) occurred when comparing the same time intervals from the fed batch. Although production of magnetite in 72 h was slightly smaller than that in fed-batch, intense production was maintained until later times (Figure 4 and Table 1), reaching 26.1 mg∙L^−1^ at 168 h. Extended exponential phase conditions and avoided conditions of late growth phases prevented the occurrence of nonmagnetic mutants. The total magnetite produced here was 104.2 mg, considering the process time and bioreactor working volume.

We only employed one dilution rate (*D* = 0.035 h^−1^), which was equivalent to 70% of the growth rate in the exponential phase (24–72 h), and one residence time. However, an expanded study testing the effects of different *D*s and longer cultivations with more residence times on magnetosome production must be performed.

## 3. Discussion

Species of *Magnetospirillum* genus require higher concentrations of oxygen for growth, and lower ones for the formation of magnetosomes [16]. For that purpose, fine process control is required to maintain oxygen concentrations in a narrow range, or culturing strategies in which the two antagonistic conditions are satisfied. In the case of the *Mv. blakemorei* strain MV-1^T^, the greatest production of magnetosomes occurs with a final electron acceptor other than oxygen, in this case, nitrous oxide [22]. The simple supplementation of this gas would already be enough to increase the production of magnetosomes without the need for complex process controls such as those described for species *Magnetospirillum* [16,18,19]. This relative simplicity increases the potential of the use of *Mv. blakemorei* strain MV-1^T^ and its magnetosomes in biotechnological applications.

Silva and colleagues [21] developed a fed-batch cultivation with solely the supplementation of iron in 24-h pulses. In that research, two supplementations were made until 120 h, with the first taking place at 72 h, when the iron level was at 30% of initial concentration. No measurement or injection of N_2_O was performed. The optimized bioreactor cultivation had magnetite productivity reaching 22.4 mg∙L^−1^ at 96 h and showed basically no changes until 192 h. Consequently, magnetite productivity decreased from 5.6 mg∙L^−1^∙day^−1^ at 96 h to 3.2 mg∙L^−1^∙day^−1^ at 192 h. In the present research, the production and productivity average of magnetosomes per cell decreased after 72 h (Figure 1a), despite N_2_O and Fe^2+^ supplementation. This dramatic decrease in magnetosomes per cell was not observed by Silva et al. [21]. The reduction of overall magnetosome production is, in part, explained by the de-acceleration of the growth rate (Figure 1a).

Regarding the role of the carbon source on magnetosome yields, different studies [21,22] show that succinate is known to sustain the heterotrophic cell growth of MV-1^T^ in microaerobic/anaerobic conditions. However, no previous research has described the kinetics of succinate consumption in MV-1^T^. Thus, it was essential to gain knowledge on succinate uptake in relation to cell growth and magnetosome formation. We also investigated succinate consumption to evaluate any eventual requirement of carbon supplementation during fed-batch. Furthermore, in chemostat cultivation, succinate monitoring ensures that carbon source concentrations are kept constant in order to support cell density. Although the carbon source was not fully depleted in our fed-batch experiments, it is known that reduced availability of carbon source causes growth de-acceleration [27]. The accumulation of toxic products and inhibitors in later phases also influences cell growth negatively [28]. In fact, genes encoding secondary metabolites with antimicrobial activity have been identified in strain MV-1^T^ [29]. Another possibility may have been the limitation of other non-measured components present in the media, such as mineral solution or specific amino acids.

There is little information on the energy consumption involved in magnetosomes’ synthesis or the physiology of MTB in general. *Mv. blakemorei* MV-1^T^ is capable of micro-aerophilic and anaerobic growth on oxygen and nitrogen oxides, respectively [22]. Despite metabolic versatility, anaerobic reduction of N_2_O by strain MV-1^T^ yields the greatest number of magnetosomes per cell among all electron acceptors [22]. In our results, we can see that the consumption profiles of Fe^2+^ and N_2_O were strongly related, with both components showing the greatest consumption between 48–72 h (Figure 1b). This coincidence might indicate a synergism between energy and material demands as Fe^2+^ was converted into Fe_3_O_4_ while N_2_O was reduced for ATP production for biomineralization. In fact, it is known that the biomineralization process is strongly dependent on energy availability [30,31]. In *Ms. magneticum* strain AMB-1, the reduction of nitrogen oxyanions provides energy necessary for magnetosome vesicle formation [30]. Furthermore, energy metabolism and magnetosome synthesis are controlled in an integrated manner at genetic level [31].

Understanding gas mass transfer kinetics is crucial for designing larger scales of a given bioprocess [32]. For continuous culture of *Mv. blakemorei*, measurement of N_2_O concentration during continuous growth is of little significance, as autoclaved fresh medium is added. Because of this, we have developed a strategy for intermittent N_2_O injection during continuous growth. Yield cultivation of *Magnetospirillum* species in bioreactors often demands strategies to keep oxygen concentrations under strict control because oxygen is required for cell growth, but anaerobiosis leads to better magnetite production [16,17,18]. Oxygen-control strategies require the online measurement of oxygen through sensitive probes and rapid monitoring of oxygen depletion due to consumption. In this sense, the strategy for sole N_2_O injection for *Mv. blakemorei* strain MV-1^T^ cultivation is simpler, because the maximum growth rate and greatest number of magnetosomes per cell occur under the same conditions.

Another advantage is that solubility of nitrous oxide in fresh and sea water is greater than that of oxygen, making N_2_O more favorable. However, this gas is not freely available in the atmosphere as oxygen is, making the gassing process more onerous. Among gases already used for bioreactor cultivation of MTB, N_2_O is the most expensive, with a cost per m^3^ twice that of argon (Table 2). In this sense, an improved injection strategy also helps diminish gassing costs. Mass transfer measurements are most commonly made for studying oxygen transfer from gas bubbles to medium in aerobic processes [33]. However, the optimization of anaerobic processes also relies on knowledge and improvement of mass transfer [34]. All strategies for the improvement of mass transfer in submerged cultures are based on the optimization of gas flow and impeller stir rate [35].

The chemostat culture reported here was developed based on the fed-batch results and represents an initial achievement, and will probably provide a valuable tool not only for magnetosome production, but also for the study of cell physiology and metabolism. Prolonged chemostat experiments will provide enough time for mutation occurrence and accumulation of mutations, and will probably generate information on experimental evolution dynamics under different cultivation conditions [40]. Particularly for MTB, MGC is highly unstable, and this instability may generate distinct subpopulations even within a single-strain culture [25]. Although we have not performed molecular studies to verify that the loss of magnetosome production was due to MGC deletions during fed-batch experiments, some hypotheses can be drafted from our results. First, after sequential generations of cultivated cells in a constantly oxygen-free environment, there may be a reduction in cell reliability on magneto-aerototaxis. The described phenomenon, combined with fluctuations in the availability of essential nutrients and final electron acceptors, seems to induce physiological states in which non-magnetic individuals are favored.

Using chemostat experimental platforms may be interesting for simulating selective pressures on biomineralization and magnetotaxis. Physiological and metabolic adaptation to culture conditions usually takes place in periods longer than those of exponential phases in batch cultures [40]. The lack of knowledge on the adaptation of MTB to rapid changes in environmental conditions and the effect of these changes in cell growth and magnetosome formation [41] could be filled by studying MTB grown in chemostats. Several studies, however, have examined the influence of chemical parameters (e.g., pH, oxygen and iron concentrations, etc.) on biomineralization and whole-cell physiology [41,42]. Those studies examined one set of predetermined conditions in cells cultivated in batch cultures. Alternatively, chemostat would provide a more in-depth mechanistic view of metabolic switches in MTB in response to controlled changes in the environmental parameters during a single cultivation experiment.

The presence of the membrane is a major advantage of magnetosomes in economic terms when compared with artificially-coated synthetic nanoparticles [15]. On the other hand, long cultivation times and milligram-scale production are limitations of the biotechnological production of magnetic nanoparticles. Batch cultures of MTB take around 50–120 h to reach cost-beneficial magnetite concentrations, compared with a few hours in chemical syntheses. In this sense, continuous culture enables microbial magnetite to be produced high at concentrations for extended periods and prevents idle time (e.g., for washing, sterilizing, lag phase) between batches.

## 4. Materials and Methods

### 4.1. Bacterial Cells

*Mv. blakemorei* strain MV-1^T^ cells were anaerobically cultivated in an optimized medium [21] in 50 mL flasks for 48 h at 28 °C before being used in fermentation experiments.

### 4.2. Bioreactor Cultivation

Volumes corresponding to a final cell concentration of 10^8^ cell/mL were inoculated in a 5-L (2-L working volume) bench bioreactor (Minifors, Infors HT–Basel, Switzerland) containing fresh growth medium. Cultivation parameters were set as follows: pH 7.0 (pH is automatically and strictly adjusted during cell growth, either in batch or fed-batch modes, by injection of sterile 1.0 N NaOH or HCl), 100 RPM stir rate, 28 °C and non-detectable oxygen. An anaerobic condition was achieved by purging sterile nitrogen and into fresh medium until the oxygen sensor reading reached zero. After that, the medium was purged with nitrous oxide (N_2_O) for 15 min. First, cultivations were carried out in fed-batch mode, generating data for continuous cultivation.

In the fed-batch (Figure A1), supplements of iron (10 mM FeSO_4_) and N_2_O (0.25 vvm) were given every 24 h, starting at the end of the exponential phase so that initial concentrations of both were re-established. One mL of the medium sample was collected every 24 h for analysis of optical density of N_2_O, iron, free nitrogen, and carbon for observation by transmission electron microscopy.

Continuous cultivation started as batch mode until it reached the exponential phase. Then the feeding of fresh medium began, simultaneously to the withdrawal of grown medium (Figure A2). The influx and efflux of medium had the same flow rate, which was determined by the exponential growth rate. In this experiment, the dilution rate, calculated as *D* = *F*/*V*, where *D* is dilution rate; *F* is volumetric flow rate (mL/h) and *V* is total medium volume (mL). *D* was equal to 70% of growth rate. FeSO_4_ (10 mM) was added to the feeding medium, whereas N_2_O was purged in intervals of 8 h for 15 min.

### 4.3. Growth Analysis

The cell density was measured by optical density at 600 nm in a spectrophotometer (Biospectro SP-22, Curitiba, Brazil). Cell concentration was obtained from the optical density (1.09 × 10^10^ cells.mL^−1^ corresponds to an OD value of 1.0). The specific growth rate (*μ*) was calculated as

*μ* = (ln *X*_2_ − ln *X*_1_)/(*t*_2_ − *t*_1_), (1)

where *X*_2_ and *X*_1_ are cell density in instants 1 and 2 and *t*_2_ − *t*_1_ is the interval between these two instants. *μ* is expressed in h^−1^.

### 4.4. Transmission Electron Microscopy

Cells and magnetosome chains were observed by transmission electron microscopy (FEI Morgagni, Hillsboro, OR, USA). The mean number of magnetosomes per cell was determined by the average number of magnetosomes in 30 cells for each sampling point. The concentration of magnetite at each time was determined by the measurement of the magnetosome diameter, which was then used to calculate its volume using the iTEM software suite (Olympus Corporation, Tokyo, Japan).

### 4.5. Nutrient Determination

Succinate was measured using high-performance liquid chromatography (HPLC-Agilent 1260, Santa Clara, CA, USA) with a chromatographic column (Aminex HPX-87H, Bio-Rad, Hercules, CA, USA) of 300 mm × 7.8 mm coupled to a refractive index detector (column temperature of 65 °C). The operating conditions were: sample volume of 20 μL, mobile phase of 0.005 M H_2_SO_4_, flow rate of 0.6 mL∙min^−1^, and column temperature of 65 °C.

The concentrations of iron and free nitrogen were determined by colorimetric methods. For the iron analysis, a kit was used (Kit Analisa 438-Belo Horizonte, Brazil), following the manufacturer’s instructions. Quantification by the kit was based on the reaction of the iron with the ferrozine reagent. For the nitrogen analysis, the colorimetric method of ninhydrin [43] was used with the final absorbance measured in a spectrophotometer (Biospectro SP-22, Curitiba, Brazil) at 575 nm.

The amount of dissolved N_2_O was analyzed by potentiometry using a specific electrode (Unisense, Aarhus, Denmark) coupled to a signal amplifier (PA2000-Unisense, Aarhus, Denmark) to read the current intensity.

The determination of mass transfer of N_2_O and O_2_ was carried out by measurements of the concentration of these gases [33]. In the first step, a volume of artificial seawater (ASW) saturated with oxygen (previously purged with compressed air) was diluted with gas-free ASW (previously boiled and vacuum-cooled) to provide 2000 mL of a solution at approximately 10% oxygen saturation. This solution was transferred to the bioreactor vessel and purged with N_2_O at a flow rate of 0.5 L∙min^−1^ under stirring rates of 100, 200, and 300 RPM. The initial concentration of O_2_ and the elapsed time to reach zero reading by the sensor were recorded. In addition, the N_2_O saturation concentration (when no increase was detected by the sensor reading) and the time required to reach it were also measured. These data were used to calculate the mass transfer coefficient of N_2_O and O_2_ as

kLa = (ln(C* − *C*_L_))/*t* (2)

where (C* − *C*_L_) refers to the variation of the concentration of each gas in the experiment. *t* is the time in minutes.

### 4.6. Gassing Costs

Gas prices have been consulted on BOC-Linde Plc (www.boconline.ie/shop, accessed on 1 May 2020). Prices were listed as of 1 May 2020.

## 5. Conclusions

The results presented here showed that in fed-batch culture, the supply of N_2_O and Fe^2+^ led to the highest production of magnetite in strain MV-1^T^ in the bioreactor (32.5 mg∙L^−1^). However, a decrease in global magnetosome production per cell and an increased number of non-magnetic cells negatively affected magnetite productivity in later cultivation phases. Due to the high demand of N_2_O for magnetosome production, and the necessity for a continuous flow of growth media, a regime for nitrous oxide injection was developed. Our pulse strategy led to an adequate supply of final electron acceptors for continuous magnetosome production. Thus, a continuous culture was designed to maintain a high activity of magnetosome formation in bacterial cells for extended periods. Despite that maximum reached production was lower than that in fed-batch, productions as high as 26.1 mg∙L^−1^ were maintained until 168 h.

The next steps of our research will aim at improving magnetite yields and, consequently, reducing the production costs of prismatic magnetite magnetosomes. Two possible strategies could be supplying other nutrients not examined here (e.g., mineral solution), and varying dilution rates in continuous culture. A deeper understanding of bacterial physiology through genome examination, focused on metabolism, will certainly benefit from chemostat culture and might help developing novel media compositions and cultivation strategies.

## 6. Patents

The results obtained in this study have been registered under the patent number BR10202001583 held in Brazil.

## Figures and Tables

**Table 1:** Maximum magnetite production and productivity and percentage of non-magnetic cells in fed batch and continuous culture.

Conduction	Time (h)	ln *X*	Magnetosomes per Cell	Non-Magnetic Cells (%)	Magnetite Production (mg∙L^−1^)	Magnetite Productivity (mg∙L^−1^∙day^−1^)
Fed batch	72	22.2 ± 0.2	12.0	15	32.5	39.7
	120	22.4 ± 0.1	6.0	45	16.1	12.6
Continuous	120	22.1 ± 0.1	10.5	13	27.1	22.7

**Table 2:** Prices and solubility of gases used in cultivations of MTB. Water solubility from literature [36,37] in a salinity of 35‰.

Gas	€/m^3^	Solubility (μmol∙L^−1^)	Works
Oxygen	4.45	182	[16]
Nitrogen	6.81	340	[38]
Argon	10.30	8.93	[39]
Nitrous oxide	20.65	19,140	[21,36]

## Data Availability

Not applicable.

## Appendix A

The experimental set-up used in this study for fed-batch and continuous culture are illustrated in Figure A1 and Figure A2 below.
